# Preparation and Performance Study of HTPB-*g*-(PNIPAM/PEG) Thermoresponsive Polymer Brush

**DOI:** 10.3390/polym16091248

**Published:** 2024-04-29

**Authors:** Pengzhi Bi, Xiuzhong Zhu, Li Tian, Jinbang Han, Wanbin Zhang, Tong Wang

**Affiliations:** 1 State Key Laboratory of Biobased Material and Green Papermaking, Key Laboratory of Pulp and Paper Science & Technology of Ministry of Education, Faculty of Light Industry, Key Laboratory for Green Leather Manufacture Technology of China National Light Industry Council, Faculty of Light Industry, Qilu University of Technology, Shandong Academy of Sciences, Jinan 250353, China; 2 Key Laboratory of Auxiliary Chemistry and Technology for Chemical Industry Ministry of Education, Shaanxi Collaborative Innovation Center of Industrial Auxiliary Chemistry and Technology, Shaanxi University of Science and Technology, Xi’an 710021, China; 3 Shandong Huatai Paper Co., Ltd. & Shandong Yellow Triangle Biotechnology Industry Research Institute Co., Ltd., Dongying 275335, China; 4 The Key Laboratory of Space Applied Physics and Chemistry, Ministry of Education and Shaanxi Key Laboratory of Macromolecular Science and Technology, School of Chemistry and Chemical Engineering, Northwestern Polytechnical University, Xi’an 710072, China

**Keywords:** hydroxyl-terminated polybutadiene, N-isopropylacrylamide, polyethylene glycol, thermoresponsive polymer brush, modification of HTPB, structural characterization, performance study

## Abstract

In recent years, a great deal of work has been devoted to the development of thermoresponsive polymers that can be made into new types of smart materials. In this paper, a branched polymer, HTPB-*g*-(PNIPAM/PEG), with polyolefin chain segments as the backbone and having polyethylene glycol (PEG) and poly(N-isopropylacrylamide) (PNIPAM) as side chains was synthesized by ATRP and click reactions using N_3_-HTPB-Br as the macroinitiator. This initiator was designed and synthesized using hydroxyl-terminated polybutadiene (HTPB) as the substrate. The temperature-responsive behavior of the branched polymer was investigated. The lower critical solution temperature (LCST) of the branched polymer was determined by ultraviolet and visible spectrophotometry (UV-vis) and was found to be 35.2 °C. The relationship between the diameter size of micelles and temperature was determined by dynamic light scattering (DLS). It was found that the diameter size changed at 36 °C, which was nearly consistent with the result obtained by UV-vis. The results of the study indicate that HTPB-*g*-(PNIPAM/PEG) is a temperature-responsive polymer. At room temperature, the polymer can self-assemble into composite micelles, with the main chain as the core and the branched chain as the shell. When the temperature was increased beyond LCST, the polyolefin main chain along with the PNIPAM branched chain assembled to form the nucleus, and the PEG branched chain constituted the shell.

## 1. Introduction

Smart materials can detect and recognize external or internal stimuli and respond to them. This stimuli-responsive behavior of these materials has opened up many avenues in the development of new materials applicable in modern high technologies. In the field of stimuli-responsive polymers, one of the most exploited stimuli is temperature, and the interest of researchers in thermoresponsive polymers has grown steadily over the years. A great deal of work has been devoted to the development of thermoresponsive polymers that can be made into new types of smart materials [1,2,3]. Poly(N-alkyl(meth)acrylamide) is one of the most extensively researched thermoresponsive polymer. Within this class, poly(N-isopropylacrylamide) (PNIPAM) has been the most investigated [4,5]. PNIPAM is a typical LCST type thermoresponsive polymer that exhibits a sharp phase transition at around 32 °C. This property is advantageous for many applications, two of which are discussed here. Firstly, it is possible to bring about essential changes in wettability, thickness, and coating morphology. Secondly, the LCST of polymers can be tuned by copolymerizing them with different monomers [6]. The LCST of PNIPAM and its copolymers are close to human physiological temperature, and thus are often used in biomedical fields, such as in biosensors [7], thermoregulatory drugs [8], gene delivery systems [9]. PNIPAM-binding proteins have also been used to regulate thermoregulatory enzyme functions [10,11]. Okano et al. [12,13] carried out studies on the utilization of PNIPAM-based polymer coatings for protein separation, cell sheet harvesting, cell isolation, and tissue engineering. They also successfully cultured hepatocyte sheets for xenotransplantation using these polymer coatings. Pei et al. [14] synthesized PNIPAM-grafted modified starch-based nanoparticles as bio-based pickering stabilizers which could be used to stabilize oil-in-water emulsions. In addition, thermoresponsive polymers of PNIPAM have also been used in wastewater treatment [15,16], oil extraction [17], and other fields.

Polymer brushes represent a special class of graft copolymers, which have side chains grafted to the main chain with a very high grafting density. Being very close to each other, the polymer chains repel each other and stretch outward to adopt a brush-like morphology at the molecular level [18]. Polymer brushes adjust their conformation in response to external stimuli (temperature, pH, ionic strength, etc.). This characteristic can be used to control surface properties in order to design smart surfaces with responsive adsorption [19], specific adhesion [20], tunable wettability [21], or antimicrobial properties [22]. Thermoresponsive polymer brushes have a unique ability to reversibly change their physicochemical properties over relatively narrow temperature ranges [23,24,25]. The key parameters that determine the properties of thermoresponsive polymer brushes include polymer density, molecular weight, and topology [26,27]. By considering and optimizing these aspects while designing, it is theoretically possible to obtain thermoresponsive polymer brushes with various properties. The majority of the current research on thermoresponsive polymer brushes has focused on PNIPAM-grafted polymer brushes [28,29,30,31,32].

Hydroxyl-terminated polybutadiene (HTPB) is one of the commonly used polyurethane matrix resins [33] that has been widely used in the defense and military field, owing to its excellent low-temperature resistance [34,35,36]. However, as compared to the applications in the defense and military fields, its applications in other fields are relatively limited. Consequently, it is crucial to expand the areas of application of HTPB through rational design. It was reported that the copolymers of HTPB would possess a low critical micelle concentration (CMC) in an aqueous solution due to its hydrophobic nature. The CMC plays an important role in the formation of micelles [37]. The introduction of HTPB during the synthesis of thermoresponsive polymer brushes on the one hand is expected to yield a polymer brush with a temperature-tunable dual self-assembly behavior, and on the other hand, could broaden the application range of HTPB. However, up until now, there have been no reports on the synthesis of the thermoresponsive polymer brushes based on HTPB as the building block with PNIPAM side chains.

**Figure 1:**
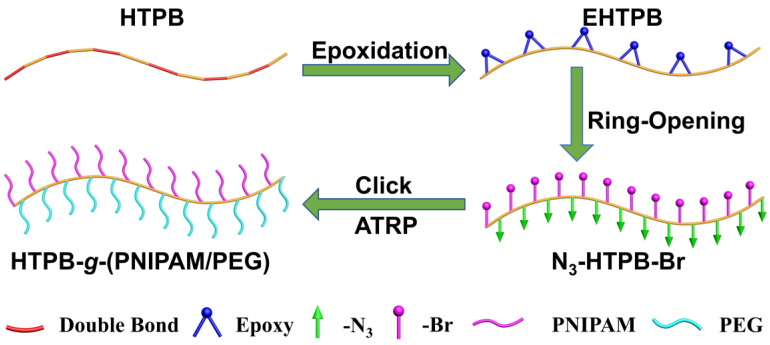
Illustration of the epoxy-modified hydroxyl-terminated polybutadiene for preparing HTPB-*g*-(PNIPAM/PEG).

Based on the above research background, firstly, epoxidation of HTPB (EHTPB) was synthesized using HTPB as the backbone reacting with 3-chloroperoxybenzoic acid. Then, a macroinitiator N_3_-HTPB-Br was obtained through ring opening for the epoxy group, and hydroxyl substitution reactions. Finally, a branched polymer (HTPB-g-(PNIPAM/PEG)) with polyolefin chain segments as the main chain and PEG and PNIPAM as side chains was designed with atom transfer radical polymerization (ATRP) and acetylene-azide “click” reaction (Figure 1). Fourier transform infrared spectroscopy (FT-IR), nuclear magnetic resonance (NMR), and size-exclusion chromatography (SEC) were used for the accurate determination of the intermediate products and the final product HTPB-*g*-(PNIPAM/PEG). Subsequently, the self-assembly behavior of the branched polymer was investigated using a combination of ultraviolet and visible spectrophotometry (UV-vis), dynamic light scattering (DLS), and other analytical techniques. We aimed to prepare a new thermoresponsive polymer brush, and in addition, provide a new strategy for the construction of temperature-responsive smart materials.

## 2. Experimental Section

### 2.1. Materials

Hydroxyl-terminated polybutadiene (HTPB, *M_n_* = 3400 g∙mol^−1^) was made in the laboratory [38]. 3-chloroperoxybenzoic acid (MCPBA, 85%) was purchased from Shanghai Aladdin Biochemical Technology Co., Ltd., Shanghai, China. Sodium azide (NaN_3_, 99%) was purchased from Vicbio (Beijing) Biotechnology Co., Ltd., Beijing, China. Ammonium chloride (NH_4_Cl, 99%), sodium bicarbonate (NaHCO_3_), N, N-dimethylformamide (DMF), methoxypolyethylene glycols (mPEG, *M_n_* = 2500 g∙mol^−1^), methanol (MeOH), diethyl ether (Et_2_O), and toluene were purchased from Sinopharm Chemical Reagent Co., Ltd., Shanghai, China. 3-bromopropyne was purchased from Sarn Chemical Technology (Shanghai) Co., Ltd., Shanghai, China. Triethylamine (TEA) and 2-bromoisobutyryl bromide were purchased from Shanghai Aladdin Biochemical Technology Co., Ltd., Shanghai, China. N-isopropylacrylamide (NIPAM, 98%) was purchased from TCI (Shanghai) Development Co., Ltd., Shanghai, China. Anhydrous magnesium sulfate (MgSO_4_) was purchased from Tianjin Kemiou Chemical Reagent Co., Ltd. (Tianjin, China). Tetrahydrofuran (THF, add sodium metal wire and benzophenone under nitrogen atmosphere before use and reflux until the system is blue, distill and collect, and use immediately) was purchased from Shanghai Macklin Biochemical Co., Ltd., Shanghai, China. Dichloromethane (DCM, add sodium metal wire and benzophenone under nitrogen atmosphere before use and reflux until the system is blue, distill and collect, and use immediately) was purchased from Sinopharm Chemical Reagent Co., Ltd., Shanghai, China. Sodium (Na) was purchased from Sinopharm Chemical Reagent Co., Ltd., Shanghai, China. Benzophenone was purchased from Shanghai Macklin Biochemical Co., Ltd., Shanghai, China. Tris(2-dimethylaminoethyl)amine (Me_6_TREN), ethanol (EtOH), copper(II) sulfate pentahydrate (CuSO_4_∙5H_2_O), isopropanol (IPA), ascorbic acid, cuprous bromide (CuBr), and cupric chloride(CuCl_2_) were purchased from Tianjin Fuyu Fine Chemical Co., Ltd., Tianjin, China. All the above reagents were analytically pure.

### 2.2. Modification of HTPB by Epoxidation with MCPBA

HTPB (0.5 g) was dissolved in 20 mL redistilled THF and added to a 50 mL round bottom flask, after which MCPBA (2.0 g) was dissolved in 15 mL dried THF and added dropwise to the above reaction solution using a constant pressure dropping funnel. The reaction was carried out at 30 °C for 6 h. After the reaction, a clear and transparent solution was obtained, part of the solvent was removed by vacuum distillation, the reaction solution was poured into EtOH, the product was washed with saturated NaHCO_3_ solution, and then washed again with EtOH, and epoxidized HTPB (EHTPB) was obtained by vacuum drying at 40 °C for 24 h.

FT-IR (KBr): 809 cm^−1^ (ν, C-O-C).

^1^H NMR (CDCl_3_, TMS): δ = 2.71 (2H, -C*H-*O-C*H*-), 2.95 (2H, -C*H-*O-C*H*-).

### 2.3. Preparation of HTPB with Hydroxyl and Azide Side Groups (N_3_-HTPB-OH)

The polymer named N_3_-HTPB-OH was prepared by the ring opening reaction of EHTPB and NaN_3_. EHTPB (0.4 g), NaN_3_ (1.114 g, 17.16 mmol), and NH_4_Cl (917.9 mg, 17.16 mmol) were dissolved in 20 mL DMF and stirred at 50 °C for 36 h. After the reaction, the filtrate was extracted, concentrated, and dissolved in 50 mL DCM, washed three times with water, and then precipitated in cold MeOH. After precipitation was repeated three times, N_3_-HTPB-OH was obtained by vacuum drying at 50 °C for 24 h.

FT-IR (KBr): 3457 cm^−1^ (ν, -OH), 2101 cm^−1^ (ν, N_3_).

### 2.4. Preparation of a Macroinitiator with Side Groups of Bromine Atoms and Azide (N_3_-HTPB-Br)

N_3_-HTPB-OH (340 mg, 3 mmol OH) was dissolved in 10 mL redistilled DCM, and dehydrated TEA (366.8 mg, 3.6 mmol) was added and stirred for 15 min at 0 °C. After that, 2-bromoisobutyryl bromide (828 mg, 3.6 mmol) was dissolved in 10 mL redistilled DCM and added dropwise to the flask with a micro-dropping funnel, controlling its dropwise acceleration, and the dropwise addition process was stirred continuously at 0 °C for 60 min. After the dropwise addition, the reaction system was sealed, and the reaction was carried out at room temperature for 20 h. After the reaction, the solvent was removed by vacuum distillation and precipitated in cold MeOH. After precipitation was repeated three times, the macroinitiator N_3_-HTPB-Br was obtained by vacuum drying at 50 °C overnight.

FT-IR (KBr): 2102 cm^−1^ (ν, N_3_), 1737 cm^−1^ (ν, C=O), 809 cm^−1^ (ν, C-O-C).

^1^H NMR (CDCl_3_, TMS): δ = 1.98 (6H, -C-(C*H*_3_)_2_Br).

### 2.5. Synthesis of Alkyne-Terminated Methoxypolyethylene Glycol (mPEG-Alk)

mPEG (2.50 g, 1 mmol), 3-bromopropyne (1.21 g, 10 mmol), and NaOH (0.40 g, 10 mmol) were dissolved in 40 mL toluene and stirred at 50 °C for 24 h. The solvent was removed by vacuum distillation and the product was dissolved in 100 mL water. The solution was extracted with DCM, the collected organic layer was dried with anhydrous MgSO_4_ and filtered, and the filtrate was concentrated by vacuum distillation and poured into 200 mL cold Et_2_O for precipitation. The precipitate was repeatedly washed three times in Et_2_O and the product was obtained by vacuum drying at 30 °C for 24 h.

FT-IR (KBr): 2101 cm^−1^ (ν, -C≡C-H), 1106 cm^−1^ (ν, C-O-C).

^1^H NMR (CDCl_3_, TMS): δ = 4.19 (2H, -C*H*_2_C*C*H), 3.65 (4H, -OC*H*_2_C*H*_2_O-), 3.38 (3H, -OC*H*_3_), 2.45 (1H, -CH_2_C*CH*).

### 2.6. Preparation of HTPB-g-(PNIPAM/PEG) Using Click Reaction and ATRP

The branched polymer HTPB-*g*-(PNIPAM/PEG) was prepared using click reaction and ATRP. The procedure was as follows: mPEG-Alk (579 mg, *M_n_* = 2500 g∙mol^−1^, 0.23 mmol) and N_3_-HTPB-Br (70 mg, 0.23 mmol) were dissolved in 4 mL DMF and added to a 25 mL Schlenk tube. Ascorbic acid (40.48 mg, 0.23 mmol) was added to the tube, and after one freeze–pump–thaw cycle, CuSO_4_∙5H_2_O (11.5 mg, 0.046 mmol) was added under argon atmosphere and the Schlenk tube was sealed with sealing film. Then, two continuous freeze–pump–thaw cycles were performed, and the reaction was carried out at 60 °C for 24 h. After the reaction, the reaction solution was fully exposed to air and stirred, and then the reaction solution was passed through a neutral alumina column with THF as eluent to remove the copper catalyst from the polymer, and the eluate was concentrated by vacuum distillation for the next reaction step. 

The concentrated product after the click reaction was used as a macroinitiator and dissolved in 3 mL mixture solvent of DMF and isopropanol (1:1), and the monomer NIPAM (3.60 g, 31.76 mmol) and the ligand Me_6_TREN (230.39 mg, 1 mmol) were added. After three continuous freeze–pump–thaw cycles, the polymerization reaction was carried out at 60 °C for 24 h. After the reaction, the reaction solution was fully exposed to air and stirred, and then the reaction solution was passed through a neutral alumina column with THF as eluent to remove the copper catalyst from the polymer, and the eluate was concentrated by vacuum distillation. The concentrated solution was loaded into a dialysis bag with the molecular weight cut-off of 5000 g∙mol^−1^ and dialyzed in DMF for 24 h to remove CuBr, CuCl_2_, and the un-reacted NIPAM monomer. After that, the dialysis bag was placed in deionized water for 48 h to replace the solvent DMF. The reaction product was obtained by removing water with a freeze dryer.

FT-IR (KBr): 3405 cm^−1^ (ν, -NH-), 1650 cm^−1^ (ν, C=O), 1554 cm^−1^ (δ, -NH-), 1115 cm^−1^ (ν, C-O-C).

^1^H NMR (CDCl_3_, TMS): δ = 4.02 (1H, -C*H*(CH_3_)_2_), 3.67 (4H, -OC*H*_2_C*H*_2_O-), 1.16 (6H, -CH(C*H*_3_)_2_).

### 2.7. Characterization of Polymers

Fourier transform infrared (FT-IR) spectra were measured using a Nicolet iS10 instrument (Thermo Fisher Scientific, Waltham, MA, USA). Using the coating method, a dichloromethane solution of a certain concentration of the polymer was prepared and applied uniformly to the potassium bromide tablets to form a film. The number of scans for a single sample was 16, and the scanning range was 450–4000 cm^−1^.

The ^1^H NMR spectra and ^13^C NMR spectra were determined using an Avance-400 NMR instrument (Bruker, Germany). CDCl_3_ was used as a solvent and TMS was used as an internal standard, and the test temperature was 25 °C.

The molecular weight (*M_n_*) and molecular weight distribution (*M_w_/M_n_*) of the polymers were determined on a Wyatt DAWN EOS SEC-MALLS (Wyatt Corporation, Goleta, CA, USA) equipped with a highly cross-linked styrene/divinylbenzene gel column (500 Å, 5 mm). The mobile phase was HPLC grade THF at a flow rate of 0.5 mL/min and the test temperature was 25 °C. The polymer solution was configured at a concentration of 15–20 mg·mL^−1^. The *dn*/*dc* values of polymer solutions were determined using an Optilab rEX type spectrophotometric refractometer (Wyatt Corporation, Goleta, CA, USA).

### 2.8. Preparation of the Micellar Solution

The micellar solution was prepared by dialysis. A total of 40 mg of polymer was dissolved in 1.5 mL DMF, and 4 mL deionized water was slowly added under magnetic stirring. After stirring for 2 h, a dialysis bag with the molecular weight cut-off of 5000 g∙mol^−1^ was selected and dialyzed in deionized water for 48 h, with water changed every 4 h. After dialysis, the retained product was prepared as a 0.5 mg·mL^−1^ concentration solution. Before measurement, the samples were kept at room temperature for at least 12 h to reach stability.

### 2.9. Study of the Self-Assembly Behaviour of the Branched Polymer in Aqueous Solution

The lower critical solution temperature was determined on a SHIMADZU UV-2550 UV-vis spectrometer (Kyoto, Japan). The concentration of the polymer aqueous solution was 1 mg·mL^−1^ and the wavelength of the test was 500 nm. Starting at 25 °C, the transmittance was recorded for every 1 °C increase. The recorded data were plotted and the temperature at which the transmittance started to drop was set to the LCST of the polymer.

The kinetic diameter (*Dz*) of polymer micelles in aqueous solution and the corresponding polymer dispersity index (PDI) were determined by DLS using a Zetasizer Nano ZS laser granulometer (Malvern, UK). The polymer solution concentration was 0.5 mg·mL^−1^, and the test was started after standing for 2 min at room temperature. The scattered light wavelength was 633 nm and the test angle was 173°.

## 3. Results and Discussion

### 3.1. Synthesis and Characterization of N_3_-HTPB-Br

**Figure 2:**
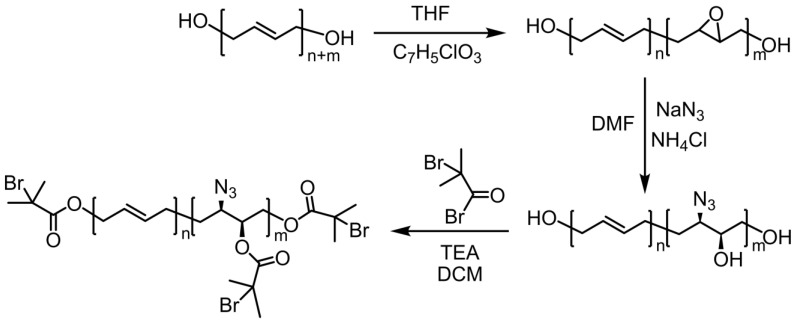
Synthetic route for preparing the comb-shaped terpolymer: N_3_-HTPB-Br.

The synthetic route for the preparation of N_3_-HTPB-Br is shown in Figure 2. EHTPB was first prepared by epoxidation of the double bonds in the main chain segment of HTPB using MCPBA. The reaction of NaN_3_ with the epoxy groups of the EHTPB yielded N_3_-HTPB-OH. Finally, the esterification reaction between 2-bromoisobutyryl bromide and N_3_-HTPB-OH was employed to prepare N_3_-HTPB-Br having reaction sites for ATRP and click reaction.

**Figure 3:**
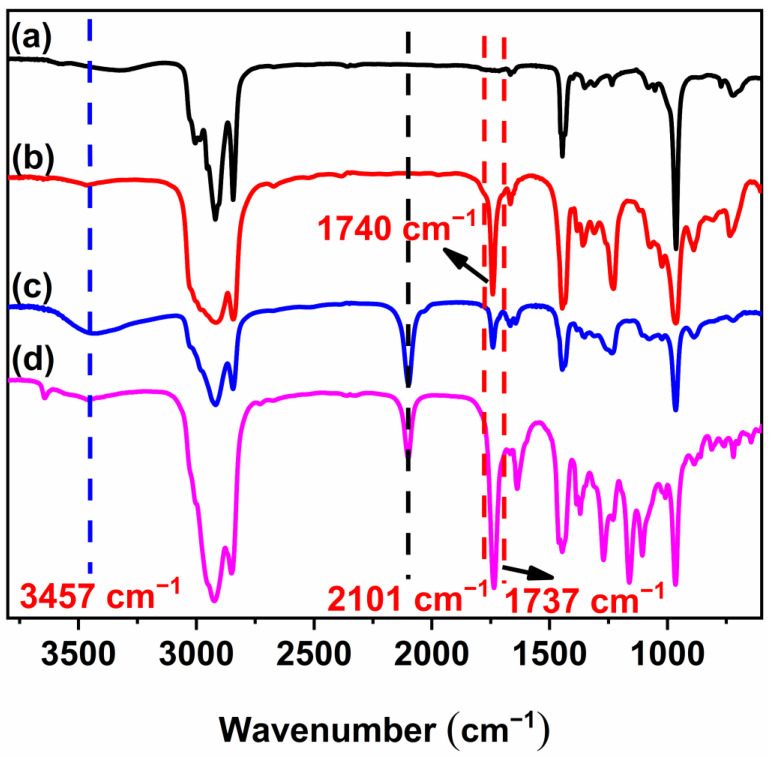
FT-IR spectra of HTPB (**a**), EHTPB (**b**), N_3_-HTPB-OH (**c**), and N_3_-HTPB-Br (**d**).

Figure 3a–d depicts the FT-IR spectra of HTPB, EHTPB, N_3_-HTPB-OH, and N_3_-HTPB-Br, respectively. Figure 3b shows the characteristic peak of the C-O-C of the epoxy group at 809 cm^−1^ and 889 cm^−1^, confirming the occurrence of the epoxidation reaction. The characteristic absorption peak at 1740 cm^−1^ could be due to the multiple epoxy groups generated by the vibrational coupling between both C-O-C and C-C. On the other hand, the characteristic peaks at 967 cm^−1^ due to the trans-1,4 double bond unit and at 724 cm^−1^ due to the cis-1,4 double bond unit indicated that only a part of the double bonds is epoxidized. The appearance of the characteristic absorption peaks of the azide group at 2101 cm^−1^ and of the hydroxyl groups at 3457 cm^−1^, as shown in Figure 3c, demonstrate the occurrence of the ring opening reaction of epoxy groups with NaN_3_. Furthermore, the appearance of a stretching vibration absorption peak of the carbonyl group at 1737 cm^−1^ and the disappearance of a peak at 3457 cm^−1^ of the hydroxyl group (Figure 3d) suggested the successful incorporation of 2-bromoisobutyryl bromide, which acts as an initiator for ATRP, into the side chains of HTPB.

**Figure 4:**
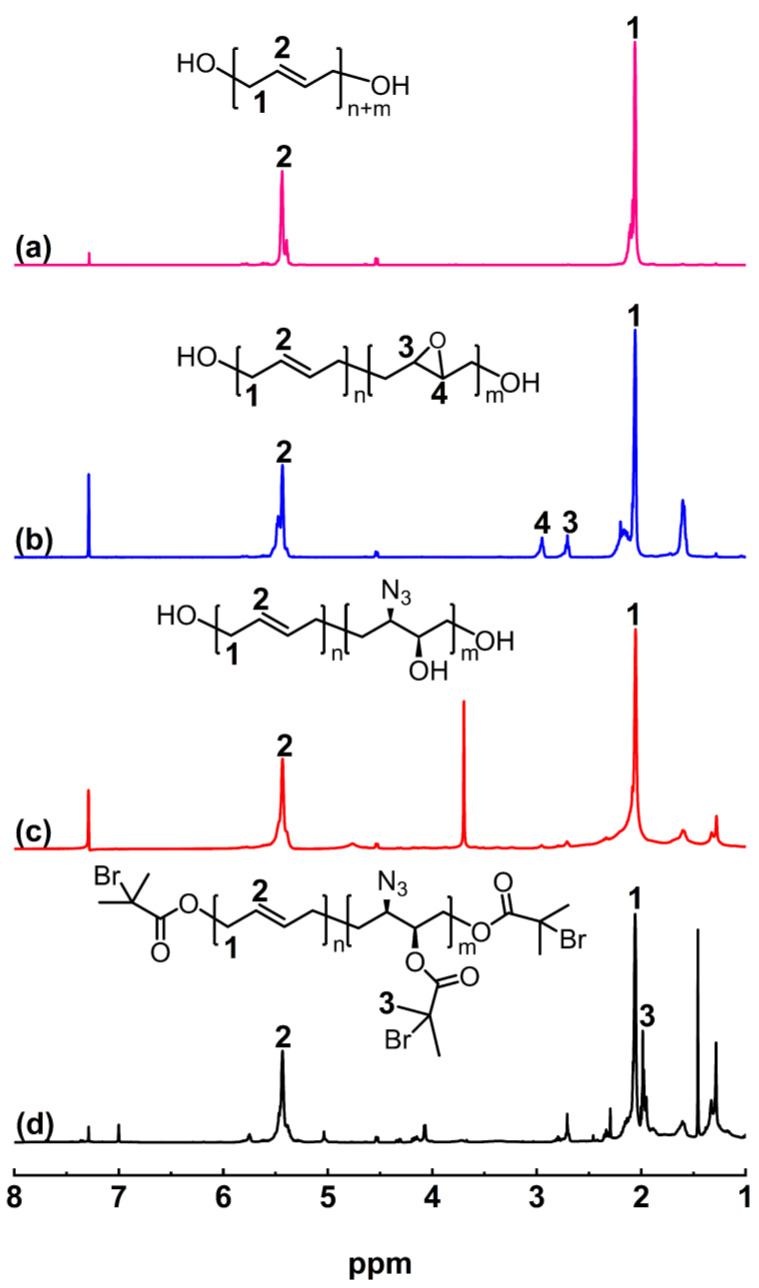
^1^H NMR spectra of HTPB (**a**), EHTPB (**b**), N_3_-HTPB-OH (**c**), and N_3_-HTPB-Br (**d**) in CDCl_3_.

Figure 4 depicts a comparison of the ^1^H NMR of HTPB, EHTPB, N_3_-HTPB-OH, and N_3_-HTPB-Br. As shown in Figure 4b, the peaks due to the epoxy group protons appear at δ = 2.68 ppm and δ = 2.92 ppm, and the integral ratio of the peak areas for these groups is approximately 1:1 (Figure S2). Furthermore, the peak area ratio of the proton peak of the epoxy groups to the proton peak of the double bonds of HTPB is about 1:5.99 (Figure S2), indicating that the epoxy groups have been successfully bonded to the molecular chain of HTPB. As shown in Figure 4c, it can be observed that the characteristic proton peak of the epoxy groups disappeared completely after the ring opening reaction with NaN_3_, indicating that all the epoxy groups in EHTPB have undergone the ring opening reaction to form azide groups and hydroxyl groups. Adding a certain amount of NH_4_Cl to the system prior to the reaction can effectively inhibit the side reactions caused by the negatively charged oxygen ions generated after the ring opening of the epoxy groups. The peak at δ = 1.96 ppm in the ^1^H NMR spectrum of N_3_-HTPB-Br corresponds to the protons of 2-bromoisobutyryl bromide. Additionally, the peak area ratio of the proton peak of the 2-bromoisobutyryl bromide to the proton peak of the double bonds of HTPB is 0.5:1 (Figure S3), indicating complete esterification and further confirming the structure of the obtained macroinitiator N_3_-HTPB-Br.

### 3.2. Synthesis and Characterization of mPEG-Alk

**Figure 5:**
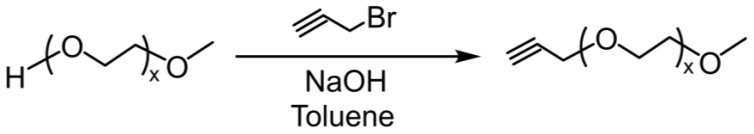
Synthesis of mPEG-Alk.

The hydrophilic segments of the branched polymer were attached to the macroinitiator via click reaction. Hydrophilic mPEG-Alk was synthesized by etherification reaction of mPEG with 3-bromopropyne using NaOH as the catalyst (Figure 5).

**Figure 6:**
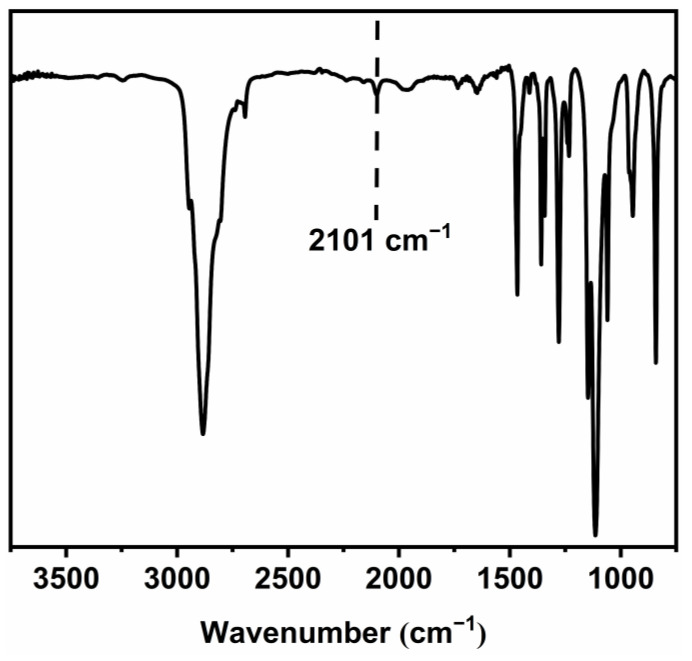
FT-IR spectrum of mPEG-Alk.

FT-IR spectrum of mPEG-Alk shows the characteristic absorption peak of the alkyne group at 2101 cm^−1^, indicating the successful synthesis of mPEG-Alk (Figure 6).

**Figure 7:**
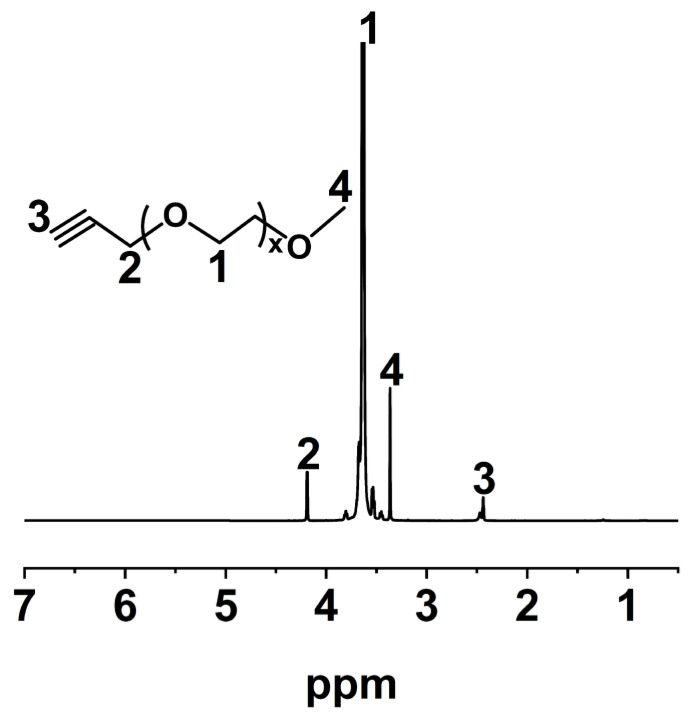
^1^H NMR spectrum of mPEG-Alk in CDCl_3_.

The ^1^H NMR spectrum of mPEG-Alk is shown in Figure 7. The peak at δ = 3.63 ppm is attributed to the methylene groups attached to the ether bonds in the main chain structure of PEG, while the peak at δ = 4.19 ppm is attributed to the methylene groups attached to the alkyne groups in the main chain structure of PEG. The proton peaks of the alkyne groups and the terminal methoxy groups in the molecular chain segment appear at δ = 2.43 ppm and δ = 3.36 ppm, respectively, with a peak area ratio of 1:3 (Figure S4), which is in agreement with the theoretical value. The above results demonstrate that all the hydroxyl groups of mPEG are replaced by the 3-propyne group resulting in the formation of mPEG-Alk.

### 3.3. Synthesis and Characterization of HTPB-g-(PNIPAM/PEG)

**Figure 8:**
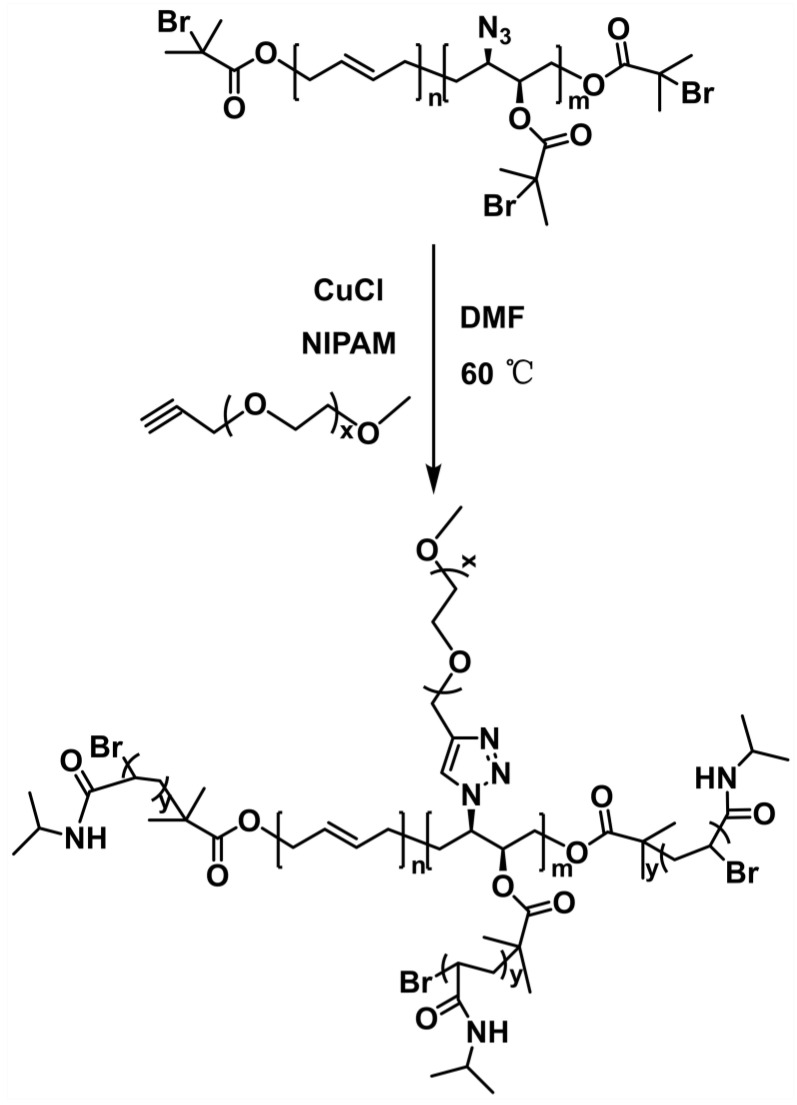
Synthesis of branched polymer: HTPB-*g*-(PNIPAM/PEG).

The synthesis of HTPB-*g*-(PNIPAM/PEG) is shown in Figure 8. Using N_3_-HTPB-Br as the macroinitiator, the thermoresponsive polymer PNIPAM was grafted to the HTPB side chains via ATRP reaction. Prior to the reaction, mPEG-Alk was added to the system to introduce the hydrophilic PEG segment in the macromolecule through the azide–alkyne click reaction. As a result, a temperature-responsive branched polymer with PEG and PNIPAM as side chains was prepared.

**Figure 9:**
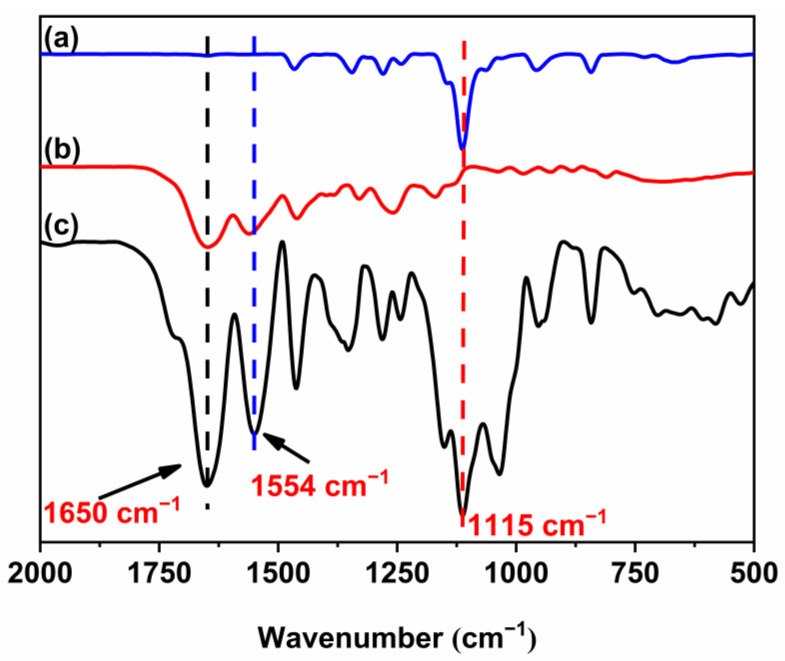
FT-IR spectra of mPEG-Alk (**a**), PNIPAM (**b**), HTPB-*g*-(PNIPAM/PEG) (**c**).

Figure 9a–c shows the FT-IR spectra of mPEG-Alk, PNIPAM, and HTPB-*g*-(PNIPAM/PEG), respectively. Successful grafting of the PNIPAM segments onto the macroinitiator was confirmed by the peaks at 3405 cm^−1^, 1650 cm^−1^, and 1554 cm^−1^, which are attributed to the stretching vibration of the N-H, stretching vibration of C=O bonds of the amide, and in-plane bending vibration of the N-H bonds, respectively. Meanwhile, the presence of the characteristic peak at 1115 cm^−1^ due to the ether bonds of PEG and disappearance of the characteristic peak at 2101 cm^−1^ due to the azide groups proves the successful occurrence of the click reaction.

**Figure 10:**
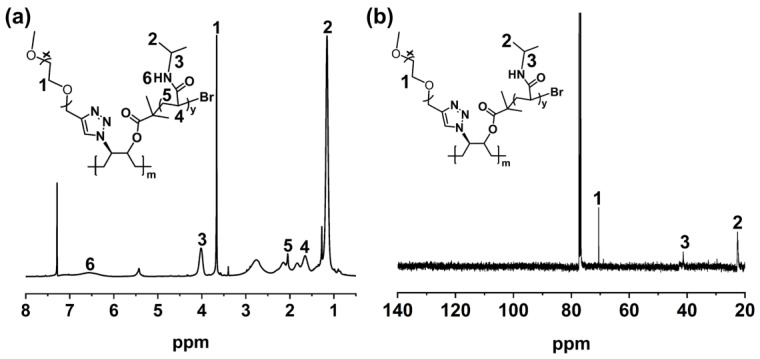
^1^H NMR (**a**) and ^13^C NMR (**b**) spectra of HTPB-*g*-(PNIPAM/PEG) in CDCl_3_.

The ^1^H NMR spectrum of HTPB-*g*-(PNIPAM/PEG) is shown in Figure 10a. It can be seen that the peak due to ethoxy group in the PEG segments appears at δ = 3.64 ppm, while the peak due to the amide bonds appears at δ = 6.53 ppm. The peaks at δ = 1.13 ppm and δ = 3.99 ppm are attributed to the methyl groups and the methylene groups on the side chains of PNIPAM, respectively, with the integral ratio of their peak areas being 6:1 (Figure S5). These results clearly indicate that the PNIPAM segments have been successfully grafted onto the macroinitiator.

The ^13^C NMR spectrum of HTPB-*g*-(PNIPAM/PEG) is shown in Figure 10b, which reveals that the characteristic peak due to the methylene groups on the main chain of PEG at δ = 70.5 ppm, the characteristic peak due to the methyl groups on the side chain of PNIPAM at δ = 22.6 ppm, and the characteristic peak of methine groups on the side chain at δ = 41.33 ppm. The above results demonstrate the successful preparation of HTPB-*g*-(PNIPAM/PEG).

**Figure 11:**
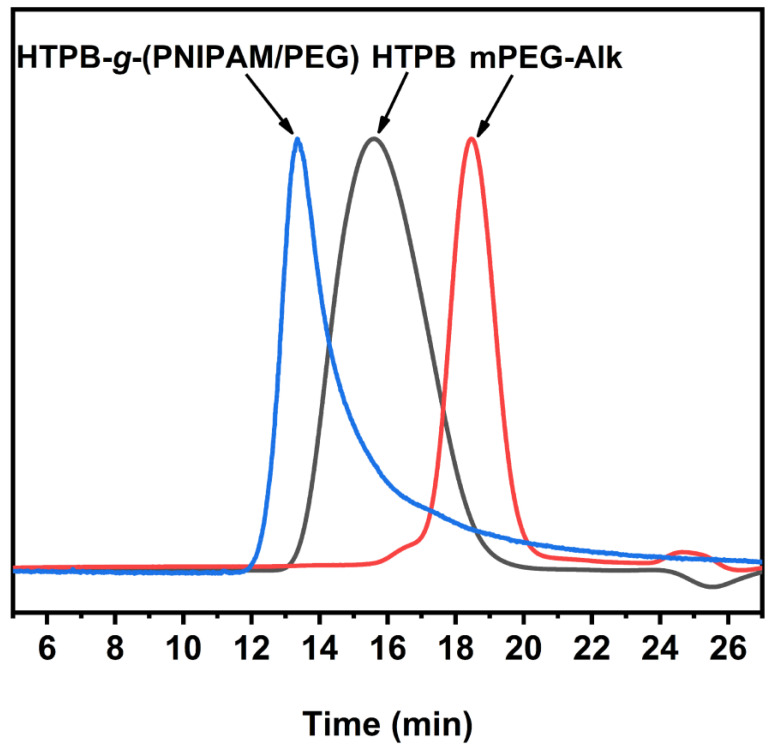
SEC traces of HTPB, mPEG-Alk, and HTPB-*g*-(PNIPAM/PEG).

The typical size-exclusion chromatography (SEC) profiles of mPEG-Alk, HTPB, and HTPB-*g*-(PNIPAM/PEG) are shown in Figure 11. From the figure it is clear that the peaks of the SEC fractions are symmetrical and sharp with a complex normal distribution, suggesting a uniform molecular weight distribution of the polymers. The peak due to the fraction of HTPB-*g*-(PNIPAM/PEG) moves towards the high molecular weight. The molecular weights (*M_n_*) of mPEG-Alk, HTPB, and HTPB-*g*-(PNIPAM/PEG) are 2500 g∙mol^−1^, 3400 g∙mol^−1^, and 185,300 g∙mol^−1^, respectively, with a molecular weight distribution of 1.19, 1.80, and 1.27, respectively. Based on the above results, it can be concluded that the branched polymer with HTPB as the main chain and PEG and PNIPAM as the branched chains was successfully prepared.

### 3.4. Self-Assembly Behaviour of the Branched Polymer in Aqueous Solution

**Figure 12:**
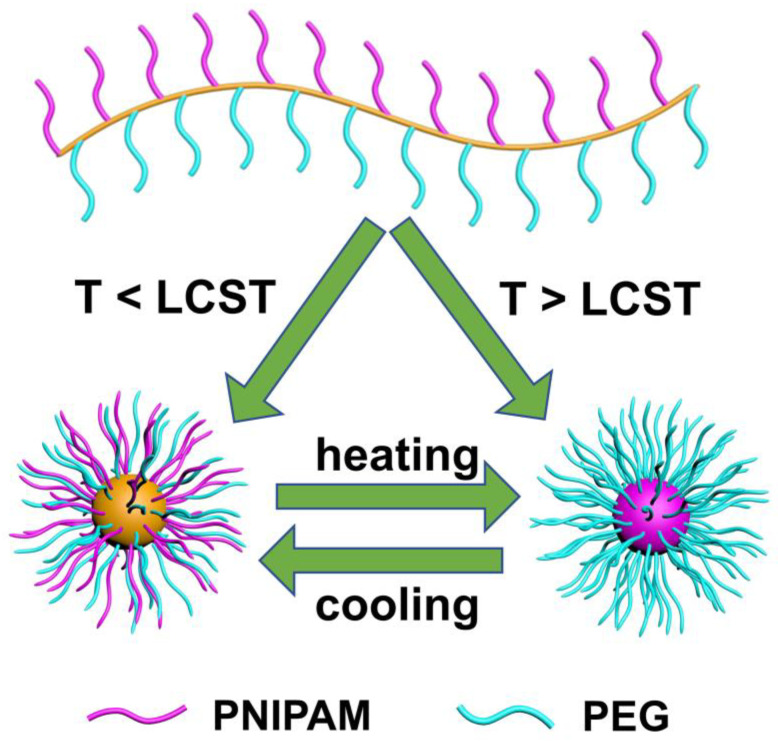
Illustration of the self-assembly behaviour of HTPB-g-(PNIPAM/PEG) in aqueous solution. The micelle with polyolefin as the nucleus and PEG and PNIPAM as the shell (left), the micelle with HTPB and PNIPAM as the nucleus and PEG as the shell (right).

PEG has good hydrophilic properties, while HTPB is a hydrophobicmaterial. Additionally, PNIPAM shows LCST in the aqueous solution. When the ambient temperature is below the LCST, PNIPAM segments can dissolve in water, whereas when it is above the LCST, PNIPAM segments show a hydrophobic behavior in water. Thus, at room temperature, owing to its amphiphilicity, HTPB-*g*-(PNIPAM/PEG) self-assembles in an aqueous solution to form micelles with polyolefin as the nucleus and PEG and PNIPAM as the shell. On the contrary, when the ambient temperature rises above the LCST, a transformation takes place in micelles, leading to further assembly to form micelles with HTPB and PNIPAM as the nucleus and PEG as the shell (Figure 12). The self-assembly behavior of branched polymer was studied using a combination of UV-vis, DLS, and other testing techniques.

### 3.5. Determination of LCST of HTPB-g-(PNIPAM/PEG) via UV-vis Spectroscopy

**Figure 13:**
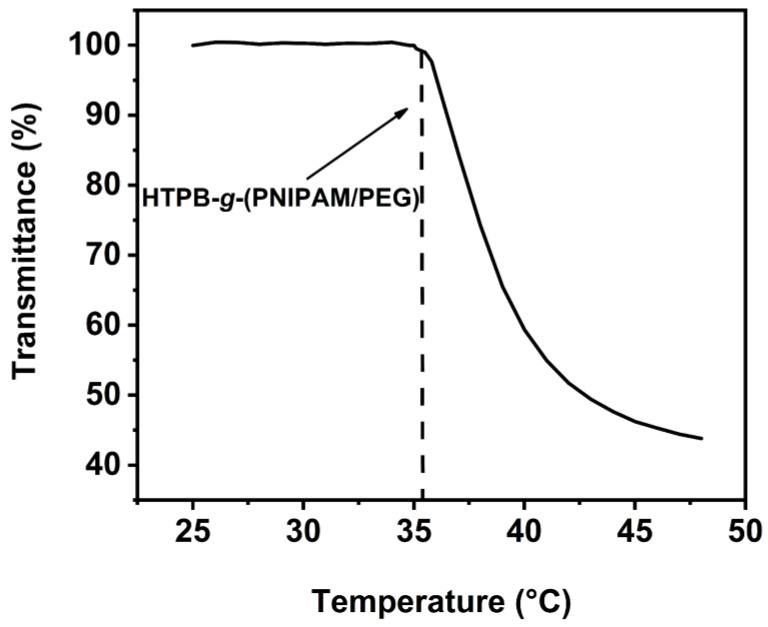
Temperature dependence of transmittance of HTPB-*g*-(PNIPAM/PEG) (1 mg·mL^−1^) during heating.

As can be seen from Figure 13, the transmittance of the solution remains essentially constant as the temperature increases. However, at a certain threshold temperature, the transmittance of the polymer solution decreases dramatically, indicating that the branched polymer is temperature responsive. The LCST of the polymer is about 35.2 °C based on the experimental data. The LCST of HTPB-*g*-(PNIPAM/PEG) is found to be higher than PNIPAM, which could be due to the presence of two branched chains of PNIPAM and PEG in the branched polymer. The hydrophilic PEG chain may influence the LCST of the polymer solution, shifting it to a higher temperature.

**Figure 14:**
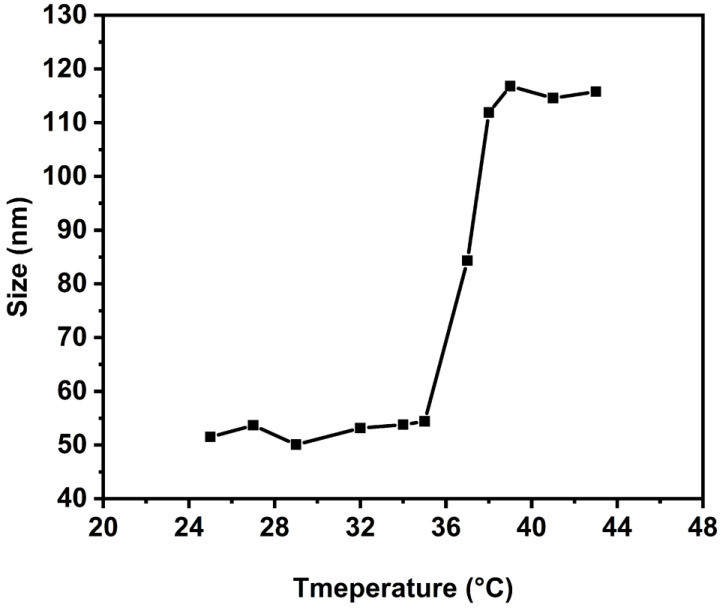
The relationship between assembly particle size (*Dz*) and temperature of HTPB-*g*-(PNIPAM/PEG) (1 mg·mL^−1^).

The self-assembly behavior of HTPB-*g*-(PNIPAM/PEG) in water was tested using DLS. The particle size of micelles shows a significant change in the diameter when the temperature is around 35 °C, and it is consistent with the LCST of the polymer determined by UV-vis (Figure 14). The increase in micelle diameter may be attributed to the fusion between the micelles. When the temperature rises above the LCST, PNIPAM undergoes a phase transition from hydrophilic to hydrophobic. Consequently, the polymer segments, initially dispersed in water, become encapsulated in the middle of the micelles. In this process, the PNIPAM segments may be involved in multiple micelle transitions, and therefore may lead to the aggregation of micelles.

**Figure 15:**
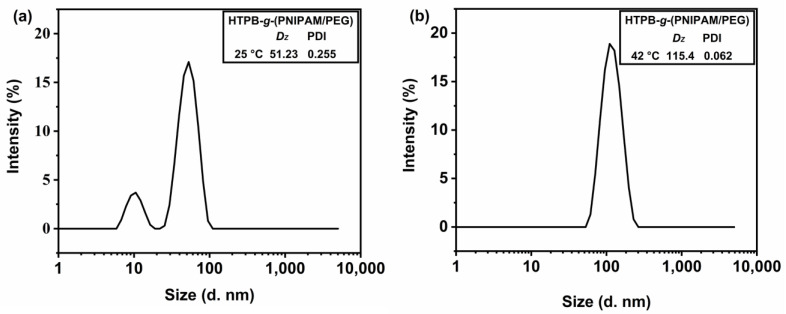
Particle size distribution of HTPB-*g*-(PNIPAM/PEG) (1 mg·mL^−1^) at 25 °C (**a**) and 42 °C (**b**) in aqueous solution.

DLS analysis of the particle size and particle size distribution of polymer micelles in an aqueous solution shows that at 25 °C, the micelle size of HTPB-*g*-(PNIPAM/PEG) is about 51 nm and the PDI is 0.255 (Figure 15a). However, at 42 °C, the particle size of micelles formed by the branched polymer becomes bigger around 115 nm, with a PDI of 0.062 (Figure 15b). Thus, it can be seen from the DLS analysis that the particle size distribution of the branched polymer exhibits a large polydispersity when the temperature is below LCST, while the self-assembly behavior of HTPB-*g*-(PNIPAM/PEG) changes and forms uniform size micelles when the temperature is increased above LCST.

## 4. Conclusions

The macroinitiator of N_3_-HTPB-Br with azide groups and bromine atoms on the side chains was successfully prepared by functionalization of HTPB. The branched polymer HTPB-*g*-(PNIPAM/PEG) with polyolefin segments as the main chain having PEG and PNIPAM as the side chains was synthesized via ATRP, and the click reaction using N_3_-HTPB-Br as the initiator and PEG and NIPAM as the building blocks. The temperature-responsive behavior of the branched polymer was investigated. The UV-vis spectroscopy was used to determine the LCST of the branched polymer and it was found to be 35.2 °C. The variation in the micelle diameter with temperature was measured by DLS, and it was found that there was a noticeable change in the micelle diameter at 36 °C, which was consistent with the result of UV-vis analysis. These findings demonstrate that HTPB-*g*-(PNIPAM/PEG) is a temperature-responsive polymer. The morphological changes of the branched polymer self-assembly were also analyzed. The analysis indicated that at room temperature, the polymer can self-assemble into composite micelles with the main chain as the nucleus and the branched chains as the shell. When the temperature was increased above LCST, changes in the hydrophobicity of the branched temperature-responsive PNIPAM occurred. This change led to the inward contraction of the molecular chain segments of PNIPAM, forming the self-assembly structure with the polyolefin main chain and PNIPAM branched chains as the nucleus and PEG branched chains as the shell. This temperature-regulated dual self-assembly polymer brush has important applications in the field of drug sustained release, and is an important material for potential drug coating and sustained release. In conclusion, we have successfully prepared a polymer brush with temperature-regulated dual self-assembly behavior using HTPB as the building block. This accomplishment provides new possibilities for the preparation and development of thermoresponsive smart materials.

## Data Availability

Data are contained within the article and Supplementary Materials.

## Supplementary Materials

The following supporting information can be downloaded at: https://www.mdpi.com/article/10.3390/polym16091248/s1, Figure S1: ^1^H NMR spectrum of HTPB in CDCl_3_. Figure S2: ^1^H NMR spectrum of EHTPB in CDCl_3_. Figure S3: ^1^H NMR spectrum of N_3_-HTPB-Br in CDCl_3_. Figure S4: ^1^H NMR spectrum of mPEG-Alk in CDCl_3_. Figure S5: ^1^H NMR spectrum of HTPB-*g*-(PNIPAM/PEG) in CDCl_3_.
