# Supplementary material for: Preparation and Performance Study of HTPB-g-(PNIPAM/PEG) Thermoresponsive Polymer Brush
Source: Polymers (Basel). 2024 Apr 29;16(9):1248. doi: 10.3390/polym16091248 (PMC11085726; doi:10.3390/polym16091248)
Supplement: Supplementary file 1 [file polymers-16-01248-s001.zip › polymers-2907944-supplementary.pdf]

Supplementary Materials

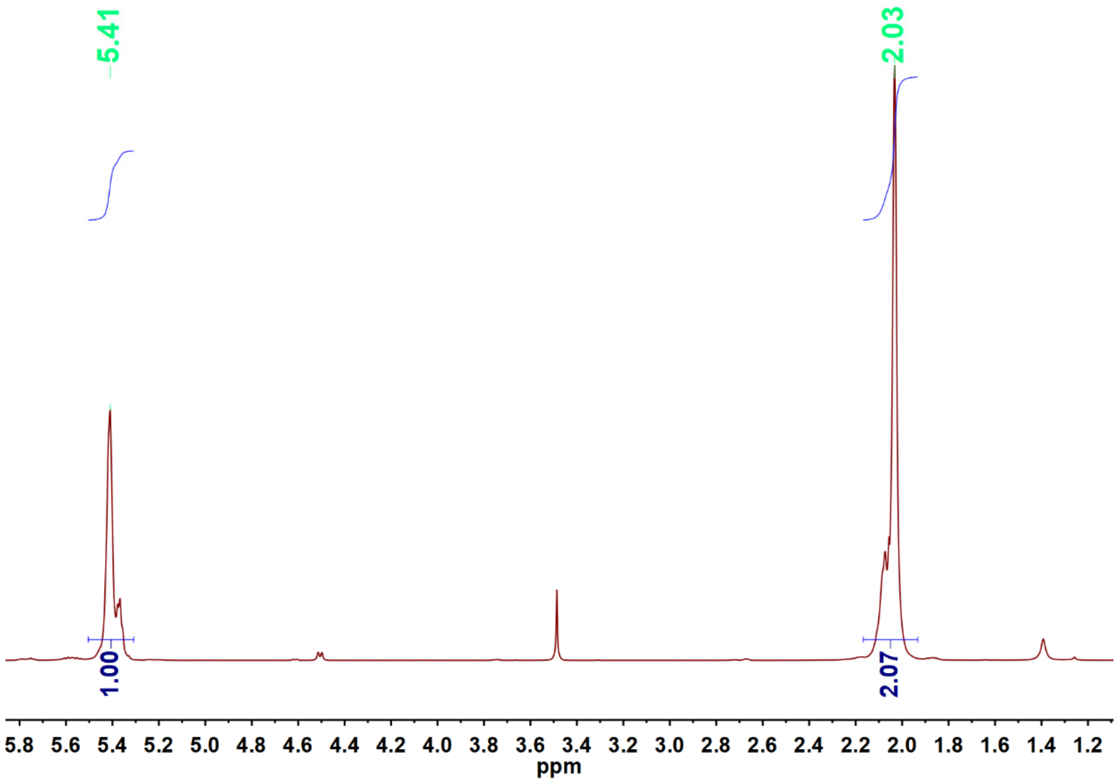

Figure S1. ^1H NMR spectrum of HTPB in $CDCl_3$.

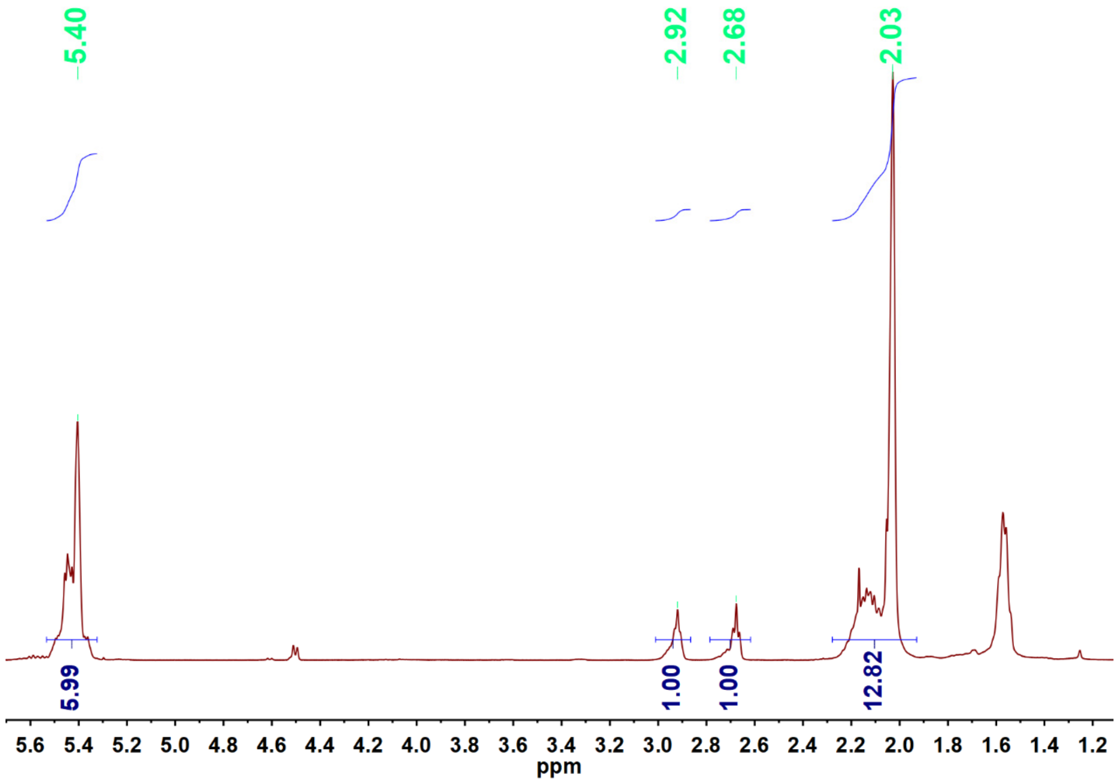

Figure S2. ^1H NMR spectrum of EHTPB in $CDCl_3$.

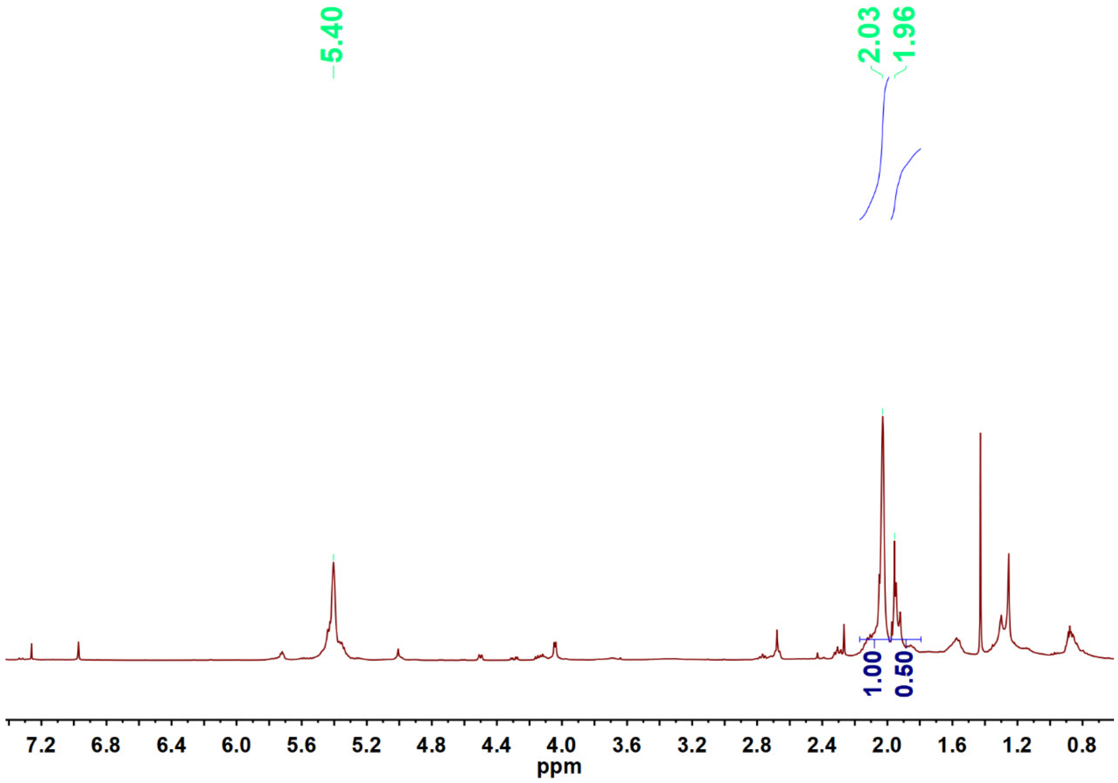

Figure S3. ^{1}H NMR spectrum of N_3-HTPB-Br in $CDCl_3$.

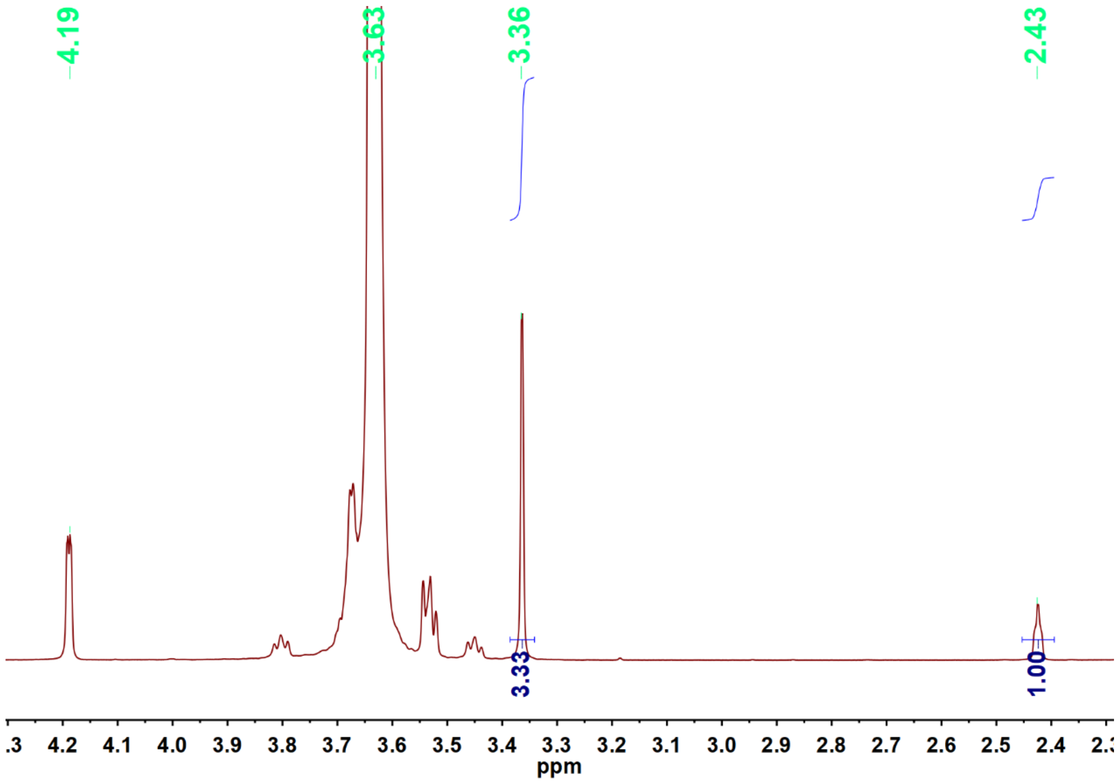

Figure S4. ^{1}H NMR spectrum of mPEG-Alk in $CDCl_3$.

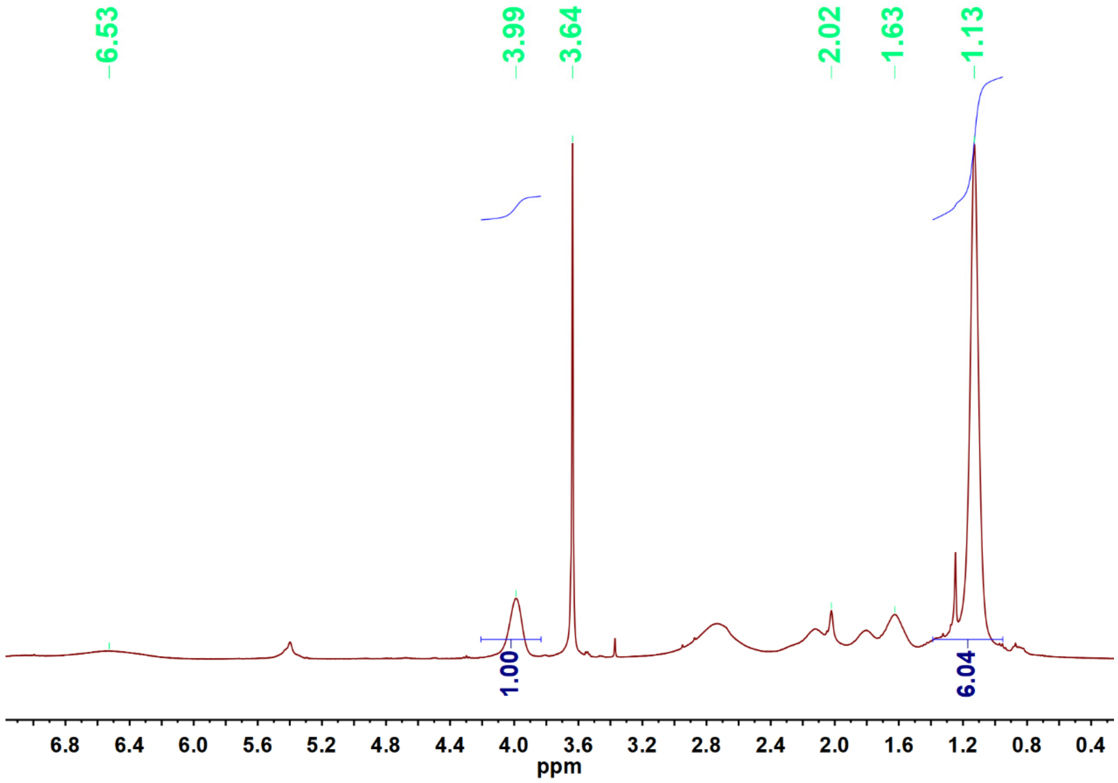

Figure S5. ^{1}H NMR spectrum of HTPB-*g*-(PNIPAM/PEG) in $CDCl_3$.
